# 肺淋巴上皮癌的免疫治疗进展

**DOI:** 10.3779/j.issn.1009-3419.2025.101.17

**Published:** 2025-10-20

**Authors:** Weizhen SUN, Yuheng ZHOU, Shoucheng FENG, Yaobin LIN, Nengqi LIN, Hao LONG

**Affiliations:** 510000 广州，中山大学肿瘤防治中心胸外科，华南肿瘤学国家重点实验室; Department of Thoracic Surgery, Sun Yat-Sen University Cancer Center, State Key Laboratory of Oncology in South China, Guangzhou 510000, China

**Keywords:** 肺淋巴上皮癌, 免疫治疗, 新辅助免疫治疗, 化免联合治疗, 肿瘤免疫微环境, Pulmonary lymphoepithelioma carcinoma, Immunotherapy, Neoadjuvant immunotherapy, Immunochemotherapy, Tumor immune microenvironment

## Abstract

肺淋巴上皮癌（pulmonary lymphoepithelioma carcinoma, PLEC）是一种罕见的与EB病毒（Epstein-Barr virus, EBV）高度相关的非小细胞肺癌亚型。目前晚期PLEC仍缺乏标准治疗方案，主要依赖以铂类为基础的化疗及放疗。近年来，随着对PLEC独特的病理特征与免疫微环境认识的深入，免疫检查点抑制剂（immune checkpoint inhibitors, ICIs）在晚期及可手术切除PLEC患者中展现了良好的临床疗效与安全性，尤其是化疗联合免疫治疗在晚期患者中显著改善了生存，新辅助免疫治疗也为可切除患者提供了降期及改善预后的新选择。然而，PLEC的罕见性限制了大规模前瞻性研究的开展，现有证据多源于回顾性分析，且免疫治疗的疗效预测生物标志物、免疫机制及最佳联合策略等关键问题仍有待阐明。本文系统综述了PLEC免疫治疗的现有临床证据、分子机制与免疫微环境特征，并对当前面临的挑战与未来的研究方向进行展望。

## Abstract (en)

Pulmonary lymphoepithelioma carcinoma (PLEC), a rare non-small cell lung cancer subtype strongly associated with Epstein-Barr virus (EBV) infection, currently lacks a standardized treatment regimen. Conventional platinum-based systemic chemoradiotherapy remains the primary approach. In recent years, its distinctive pathological features and tumor immune microenvironment have established a compelling rationale for immune checkpoint inhibitors (ICIs). Clinical studies have demonstrated promising efficacy and acceptable safety of ICIs, both as monotherapy and in combination with chemotherapy, in patients with advanced and resectable PLEC. Neoadjuvant immunotherapy has also emerged as a viable strategy to downstage tumors and improve resectability in operable cases. However, the rarity of PLEC has limited the availability of large-scale prospective trials, and current evidence is predominantly derived from retrospective analyses. Key challenges, including predictive biomarkers, mechanisms of immunotherapy, and optimal combination strategies, remain to be addressed. This review systematically summarizes the current clinical evidence, molecular mechanisms, and immune microenvironment characteristics of PLEC, and discusses future research directions.

肺淋巴上皮癌（pulmonary lymphoepithelioma carcinoma, PLEC）是一种极其罕见的非小细胞肺癌（non-small cell lung cancer, NSCLC）亚型，占所有肺癌病例数不足1%^[1]^，于1987年首次被Begin等^[2]^报道。该病主要发生在亚洲地区不吸烟的年轻人群中，西方人群发病率极低^[3]^；它的病因和诊断与EB病毒（Epstein-Barr virus, EBV）感染密切相关^[4]^，肿瘤组织中常检测到EBV编码的小RNA（EBV-encoded RNA, EBER）。且有研究^[5]^发现，PLEC有着与未分化鼻咽癌类似的病理特征。尽管2021年世界卫生组织将PLEC归类为一种特殊类型的肺鳞状细胞癌^[6]^，但由于其数量稀少和独特的临床病理特征，人们对其的认识仍然不足，这一分类标准仍存争议。目前尚无晚期PLEC的标准治疗方案。

大多数PLEC患者在早期发现，根治性切除是首选治疗方法^[7]^，与其他类型NSCLC患者相比有更好的预后^[8,9]^。然而，晚期PLEC的预后较差，其中位生存期约为24个月^[10]^。由于PLEC中很少检测到表皮生长因子受体（epidermal growth factor receptor,* EGFR*）突变、c-ros肉瘤致癌因子-受体酪氨酸激酶（ROS proto-oncogene 1, receptor tyrosine kinase,* ROS1*）突变和间变性淋巴瘤激酶（anaplastic lymphoma kinase, *ALK*）重排等驱动突变^[11,12]^，靶向治疗对于晚期PLEC效果不佳。目前晚期PLEC患者通常采用以吉西他滨联合铂类为主的化疗和放疗等全身治疗方案^[13-15]^。研究^[16]^证实，铂类化疗是晚期PLEC总生存期（overall survival, OS）的独立预测因素，其中吉西他滨联合铂类的方案展现出相对更优的疗效。一项回顾性研究^[17]^纳入了127例分别接受吉西他滨加铂类、紫杉烷加铂类或培美曲塞加铂类一线治疗的不可切除PLEC患者，显示吉西他滨加铂类组的中位无进展生存期（progression-free survival, PFS）显著优于紫杉烷加铂类组和培美曲塞加铂类组（8.8 *vs* 7.9 *vs* 6.4个月，*P*=0.031）。此外，PLEC对放疗表现出较高的敏感性。既往研究^[15]^显示，其客观缓解率（objective response rate, ORR）可达82.4%，显著高于其他NSCLC亚型（约28.5%）。一项回顾性研究^[15]^纳入103例III期N2的PLEC患者，并将其分为G1根治性放化疗组、G2根治性手术+辅助放化疗组、G3根治性手术+辅助化疗组，结果显示G1组和G2组的PFS（3年PFS率：76.7% *vs* 85.7% *vs* 47.6%）和OS（3年OS率：90.9% *vs* 100.0% *vs* 88.8%）均显著优于G3组，而G1组和G2组的PFS、OS无明显差异（*P*>0.05），证实放疗在改善局部控制和生存中的优异疗效。

随着免疫时代的到来，免疫检查点抑制剂（immune checkpoint inhibitors, ICIs）已彻底改变晚期NSCLC的治疗格局。多项大型前瞻性临床研究^[18-21]^（如KEYNOTE、CheckMate系列）已证实ICIs可显著延长患者OS和PFS，并发现程序性死亡配体1（programmed death ligand 1, PD-L1）表达水平和肿瘤突变负荷（tumor mutational burden, TMB）是预测其疗效的重要生物标志物。随着研究^[22,23]^发现，与肺鳞状细胞癌或肺腺癌患者相比，PLEC的PD-L1的表达水平明显更高，提示免疫治疗可能对PLEC患者有显著疗效。近年来，已有数项研究在探索免疫治疗在晚期PLEC患者中的疗效和安全性，并取得了重要突破。本文就PLEC的免疫治疗进展进行了综述，以期对PLEC的治疗选择提供参考。

## 1 PLEC与EBV

EBV是一种广泛分布的人类疱疹病毒，感染了全球95%以上的人口，导致1.8%的恶性肿瘤死亡^[24]^。通过原位杂交检测EBER发现，超过80%的PLEC亚洲患者肿瘤细胞中存在EBV感染，而正常肺组织及肺癌其他亚型中极少检出^[25,26]^。

近年来，鉴于PLEC与EBV感染高度相关性，已有多项研究^[4,27-31]^发现血浆EBV DNA浓度可作为PLEC疾病进展、预后预测及疗效监测的生物标志物。Xie等^[4]^回顾性分析了中国5家医院429例I-IV期PLEC患者，显示晚期（III/IV期）、淋巴结转移、淋巴血管侵犯患者的EBV DNA水平显著升高（*P*<0.001），提示EBV DNA浓度与PLEC进展密切相关；且在后期随访中发现EBV DNA高拷贝数（≥4000拷贝/mL）与较短的OS（HR=3.67, *P*<0.001）和无病生存期（disease-free survival, DFS）显著相关（*P*<0.001），根治术后3个月可持续检测到EBV DNA的患者OS和DFS同样更差（*P*<0.001）。研究^[27-31]^同样证实高浓度EBV DNA与PLEC患者的预后较差有关，其中Zhang等^[31]^发现基线EBV DNA≥1000拷贝/mL的患者PFS和OS较差（HR=2.05, *P*<0.001）。另一多中心回顾性研究^[30]^发现基线EBV DNA≥2000拷贝/mL患者PFS和OS显著更差（HR=1.97和2.95, *P*<0.001）。近期一项前瞻性研究^[32]^也显示基线EBV DNA≥1500拷贝/mL与较差PFS有关（HR=2.30, *P*=0.045）。且研究^[4,27]^发现血浆EBV DNA升高平均早于临床进展约4个月，可用于早期识别患者复发。然而，现有证据多源于回顾性研究，部分存在样本量有限、数据完整性不足等固有局限。此外，EBV感染存在地区差异。有研究^[33]^发现，EBV阳性PLEC患者主要集中在东南亚地区，而西方PLEC患者大多与EBV无关。同时，目前尚缺乏针对EBV不同状态（阳性 *vs* 阴性）患者免疫治疗应答差异的系统性比较。因此，EBV在PLEC发生发展中的确切作用及其预测价值，仍需通过多中心前瞻性研究进一步验证。

## 2 PLEC免疫微环境

PLEC的免疫微环境在肿瘤的发生、发展及治疗中发挥着重要作用，其特征包括持续的EBV感染、丰富的淋巴细胞浸润以及PD-L1的高表达。

### 2.1 免疫细胞构成及炎症反应

淋巴细胞和单核细胞是参与肿瘤相关炎症的主要免疫细胞类型，已被证明与多种恶性肿瘤的预后相关^[34-36]^。PLEC肿瘤间质及癌巢内存在大量的肿瘤浸润淋巴细胞（tumor infiltrating lymphocytes, TILs），其中以CD8^+^细胞毒性T细胞为主^[37,38]^。Yeh等^[39]^通过CD45免疫染色量化28例PLEC患者肿瘤内淋巴细胞浸润程度，发现CD45高表达组与肿瘤体积较小、最大标准摄取值（maximum standardized uptake value, SUVmax）较低、无复发生存期（relapse-free survival, RFS）延长相关，提示淋巴细胞浸润可能与抗肿瘤免疫相关。此外，与其他NSCLC亚型相比，PLEC肿瘤细胞可特异性表达单核细胞趋化蛋白-1（monocyte chemoattractant protein-1, MCP-1），从而吸引肿瘤相关巨噬细胞（tumor-associated macrophages, TAMs）浸润^[40]^，形成有利于EBV存活的微环境。Wang等^[41]^回顾性分析74例PLEC患者的外周血单核细胞/淋巴细胞比值（monocyte-to-lymphocyte ratio, MLR），发现MLR是PLEC患者OS、PFS的独立预后因素，高MLR（≥0.262）与较差的OS和PFS显著相关，这进一步支持了高淋巴细胞浸润与患者预后改善相关的观点。且既往研究^[42]^发现循环单核细胞增多可能促进肿瘤微环境中TAMs的生成，进而促进肿瘤的进展与转移。相反，淋巴细胞可能在宿主抗肿瘤免疫中发挥重要作用，从而有助于控制肿瘤生长和消除残留病灶。

### 2.2 PD-L1表达及其调控机制

63.3%-75.8%的PLEC患者肿瘤细胞中PD-L1表达阳性，显著高于其他NSCLC亚型^[43-46]^。既往研究^[47-49]^发现，PD-L1的高表达与EBV感染密切相关。研究^[48,50]^显示，EBV可通过多种机制上调PD-L1表达：（1）潜伏膜蛋白1（latent membrane protein 1, LMP1）的核心驱动作用：EBV编码的LMP1被认为是调控PD-L1最重要的病毒癌蛋白，其作用类似于组成性激活的CD40受体，通过激活Janus激酶/信号转导及转录激活因子3（Janus kinase/signal transducer and activator of transcription 3, JAK/STAT3）、丝裂原活化蛋白激酶/激活蛋白-1（mitogen-activated protein kinases/activator protein-1, MAPKs/AP-1）、核因子κB（nuclear factor-κB, NF-κB）信号通路直接诱导PD-L1转录；（2）干扰素-γ（interferon-γ, IFN-γ）的间接诱导作用：EBV感染导致的慢性炎症微环境会促进IFN-γ分泌，激活JAK2/STAT1/干扰素调节因子1（JAK2/STAT1/interferon-regulatory factor 1, JAK2/STAT1/IRF-1）通路直接结合PD-L1启动子并与LMP1激活的磷脂酰肌醇3-激酶/蛋白激酶B/哺乳动物雷帕霉素靶蛋白（phosphatidylinositol 3-kinase/protein kinase B/mammalian target of rapamycin, PI3K/PKB/mTOR）通路产生协同效应，共同维持PD-L1的基础和高水平表达；（3）EBV非编码RNA的微调机制：EBV编码的微小RNA（如miR-BHRF1-2-5p）可通过靶向宿主肿瘤蛋白p53（tumor protein p53, *TP53*）基因或直接抑制微小RNA-34a（microRNA-34a, miR-34a）解除对PD-L1 mRNA的翻译抑制。EBV核抗原1（Epstein-Barr nuclear antigen 1, EBNA1）可通过上调JAK2增强IFN-γ信号通路，EBNA2则通过招募转录因子至miR-34a启动子，下调PD-L1的负调控因子，从而间接上调PD-L1表达。

### 2.3 免疫逃逸和细胞互作

PLEC的免疫微环境中，EBV感染诱导的PD-L1上调与T细胞功能耗竭密切相关。研究^[51]^表明，T细胞表面表达的程序性细胞死亡蛋白1（programmed cell death protein 1, PD-1）受体与肿瘤细胞上PD-L1配体的结合，可激活下游抑制性信号通路，进而抑制T细胞功能。该结合过程促使含Src同源区2的蛋白酪氨酸磷酸酶-2（src homology-2 domain-containing protein tyrosine phosphatase-2, SHP-2）被募集至PD-1胞内段的免疫受体酪氨酸抑制基序（immunoreceptor tyrosine-based inhibitory motif, ITIM）和免疫受体酪氨酸转换基序（immunoreceptor tyrosine-based switch motif, ITSM），引起包括PI3K/PKB、RAS/MAPK及JAK/STAT等关键信号通路中相关分子的去磷酸化。最终，这一机制削弱了T细胞的细胞因子分泌、细胞毒性作用以及增殖与存活能力，从而推动T细胞进入功能耗竭状态。此外，TAMs可通过分泌免疫抑制性细胞因子如白细胞介素-10（interleukin-10, IL-10）和转化生长因子-β（transforming growth factor-β, TGF-β），进一步削弱T细胞的抗肿瘤免疫应答，形成抑制性免疫微环境^[52,53]^。

尽管PD-L1高表达抑制了T细胞活性，增强了肿瘤的免疫逃逸能力，但也提示了ICIs的治疗潜力。目前多数研究^[30,32,54-57]^支持PD-L1作为PLEC免疫治疗疗效的预测标志物。多项回顾性研究^[30,54-57]^分析显示，PD-L1阳性（≥1%）和PD-L1高表达（≥50%）的患者PFS显著延长，如Zhong等^[55]^发现PD-L1≥1%的患者PFS（8.4 *vs* 2.1个月，*P*=0.015）和OS（26.0 *vs* 4.6个月，*P*<0.001）显著延长。但其中部分研究^[57]^未观察到OS的显著改善。同时，亦有研究（包括近期一项前瞻性研究）^[26,32]^报道PD-L1表达水平与PFS和OS之间缺乏明确相关性。这些不一致的结果提示，PD-L1的预测价值仍需更多前瞻性研究去进一步证实，以明确其在PLEC中的临床指导地位。

## 3 PLEC的免疫治疗临床进展

基于PLEC独特的免疫微环境，ICIs在其治疗中的应用已成为研究热点。现有临床研究主要聚焦于晚期患者的免疫治疗（包括单药及联合化疗）以及可手术患者的新辅助免疫治疗。

### 3.1 晚期PLEC的免疫治疗进展

近年来，多项回顾性研究^[30,31,54-60]^探讨了免疫治疗在晚期PLEC中的疗效和安全性。2021年，Fu等^[59]^首次回顾性比较了27例转移性或复发性PLEC患者接受免疫治疗（联合或不联合化疗）和单独化疗的疗效。结果显示，免疫治疗组的中位PFS显著优于化疗组（15.0 *vs* 7.9个月，*P*=0.005），且未报告严重药物不良反应。该研究表明免疫治疗在晚期PLEC中安全可行、预后良好，并初步探索了免疫联合化疗策略的疗效。随后，多项回顾性研究^[31,55-58]^探索了晚期PLEC免疫单药治疗和化免联合治疗的效果。一项多中心研究^[56]^纳入了68例晚期（IIIB/IV期）PLEC患者，分别接受化疗、免疫单药及化免联合治疗。结果显示，化免联合治疗组、免疫单药组及化疗组的中位PFS分别为11.8、11.0及6.9个月；与化疗组相比，化免联合治疗组与更好的2年PFS显著相关（HR=0.38, 95%CI: 0.18-0.78, *P*=0.007）。但由于该研究免疫治疗组和化免联合治疗组样本量较小（*n*=7和*n*=12），统计效力可能会受到影响。Zhang等^[31]^回顾性分析了133例晚期（IIIB-IV期）PLEC患者接受化免联合治疗或化疗的疗效和安全性，结果与既往研究相当，联合治疗组的中位PFS（12.8 *vs* 7.7个月，HR=0.48，*P*=0.001）和ORR（74.5% *vs* 34.6%, *P*<0.001）均显著高于化疗组，且联合治疗组较化疗组OS趋势改善（中位OS：未达到 *vs* 35.7个月，*P*=0.065），安全性良好，提示免疫治疗可能有更好的生存获益。随后，另一项单中心回顾性研究^[55]^进一步探索了转移性PLEC患者接受化免联合治疗与免疫单药治疗的疗效对比。研究显示，联合治疗组的ORR（27.8% *vs* 13.3%）与中位PFS（10.1 *vs* 7.7个月）均优于单药组，但联合治疗组中位OS较短（19.7 *vs* 24.9个月），这可能与联合治疗组样本量较小（*n*=18）及研究随访截止时部分患者尚未达到终点（13例仍在治疗中）导致数据未完全成熟有关。后来Pang^[54]^、Lei^[60]^和Yang^[30]^的研究进一步证实了化免联合治疗在晚期PLEC中的生存获益，并且发现化免联合治疗对于二线治疗患者仍有更好的疗效。其中Yang^[30]^的研究纳入了目前最多的患者例数（*n*=252），并且有着长期随访数据，发现化免联合治疗组的中位OS显著优于化疗组（26.1 *vs* 19.2个月，HR=0.41，*P*<0.001）。近期，由Lin等^[32]^牵头完成的国际首项前瞻性研究，对晚期PLEC的免疫治疗策略进行了探索。该研究为一项单臂、多中心、II期临床研究，纳入30例未经治疗的IIIB-IV期PLEC患者，接受信迪利单抗联合吉西他滨与卡铂一线治疗。研究结果显示，该联合方案疗效显著，整体ORR达到了73.3%，疾病控制率（disease control rate, DCR）达100%，中位PFS为13.5个月（95%CI：12.2-24.5个月），中位OS为46.9个月（95%CI：29.5个月-未达到）。此项研究证实，信迪利单抗联合吉西他滨和卡铂在晚期PLEC患者中展现出显著抗肿瘤活性，尽管≥3级治疗相关不良事件（treatment-related adverse events, TRAEs）为70%，但均是临床管理较为成熟的中性粒细胞减少、贫血等血液学毒性，整体安全性可控。

这些研究大量填补了PLEC免疫治疗领域的证据空白，并证实了化免联合方案在延长晚期PLEC患者生存期的显著获益及其可控的安全性。尽管大部分为回顾性研究存在其固有局限，但结果仍为临床决策提供了重要的参考价值（表1^[28,30-32,54-60]^）。

**表1:** 既往PLEC免疫治疗文献

Study	Study type	Disease stage	Number of patients	Treatment regimen	ORR	mPFS (mon)	mOS (mon)
Fu, 2021^[59]^	Single-center retrospective	Metastatic or recurrent	27	Immunothrapy group (*n*=10) *vs* Chemotherapy group (*n*=17)	80.0% *vs* 70.5%	15.0 *vs* 7.9	NS
Wu, 2021^[58]^	Single-center retrospective	Advanced	5	Immunothrapy group (Sintilimab, Pembrolizumab, Nivolumab)	40.00%	NS	NS
Xiao, 2022^[56]^	Multi-center retrospective	Stage IIIB-IV	68	Immunochemotherapy group (*n*=12) *vs* Immunotherapy group (*n*=7) *vs* Chemotherapy group (*n*=49)	33.3% *vs* 28.6% *vs* 61.2%	11.8 *vs* 11.0 *vs* 6.9	NS
Zhang, 2022^[31]^	Multi-center retrospective	Stage IIIB-IV	133	Immunochemotherapy group (*n*=55) *vs* Chemotherapy group (*n*=78)	74.5% *vs* 34.6%	12.8 *vs* 7.7	NS *vs* 35.7
Zhong, 2022^[55]^	Single-center retrospective	Metastatic	48	Immunochemotherapy group (*n*=18) *vs* Immunotherapy group (*n*=30)	27.8% *vs* 13.3%	10.1 *vs* 7.7	19.7 *vs* 24.9
Zhou, 2022^[57]^	Multi-center retrospective	Locally advanced or metastatic	36	Immunotherapy or combined with chemotherapy/anti-angiogenesis	57.60%	17.2	NS
Pang, 2023^[54]^	Single-center retrospective	Stage IIIA-IV	96	First-line: Immunochemotherapy group (*n*=49) *vs* Chemotherapy group (*n*=45); Second-line: Immunochemotherapy group (*n*=13) *vs* Immunothrapy group (*n*=32)	67.4% *vs* 53.3%；38.5% *vs* 9.4%	15.6 *vs* 8.6; 21.7 *vs* 7.8	NS
Lei, 2025^[60]^	Single-center retrospective	Stage II-IIIB	72	Immunochemotherapy group (*n*=24) *vs* Chemotherapy group (*n*=48)	75.0% *vs* 58.3%	NS *vs* 35	NS
Yang, 2025^[30]^	Multi-center retrospective	Stage IIIB-IV	252	First-line: Immunochemotherapy group (*n*=83) *vs* Immunotherapy group (*n*=36) *vs* Chemotherapy group (*n*=133); Second-line: Immunochemotherapy group (*n*=28) *vs* Immunotherapy group (*n*=26) *vs* Chemotherapy group (*n*=194)	NS	17.6 *vs* 14.5 *vs* 8.7; 5.1 *vs* 4.3 *vs* 3.3	26.1 *vs* 23.1 *vs* 19.2；13.5 *vs* 10.7 *vs* 8.9
Chen, 2025^[28]^	Single-center retrospective	Advanced	190	Immunochemotherapy group (*n*=88) *vs* Chemotherapy group (*n*=102)	71.1% *vs* 42.2%	16.5 *vs* 7.2	NS *vs* 44.4
Lin, 2025^[32]^	Single-arm, multi-center, II	Stage IIIB-IV	30	Sintilimab+Gemcitabine+ Carboplatin	73.30%	13.5	46.9

PLEC: pulmonary lymphoepithelioma carcinoma; ORR: objective response rate; mPFS: median progression-free survival; mOS: median overall survival.

### 3.2 早中期PLEC的新辅助免疫治疗进展

新辅助免疫治疗旨在通过术前治疗缩小肿瘤体积，降低肿瘤分期并消除微转移，从而提高手术切除率并改善预后。近年来，新辅助免疫治疗在NSCLC中展现出良好的应用前景，但在PLEC中的应用仍需进一步探索。

2023年，Hong等^[61]^开展的研究为此提供了初步证据。该研究纳入39例I-III期PLEC患者，分别接受新辅助免疫（联合或不联合化疗）或单纯化疗。结果显示，免疫组和化疗组分别有18例（56.25%）和3例（42.9%）患者出现淋巴结降期；新辅助治疗后免疫组和化疗组分别有27例（84.4%）和7例（100%）的患者实现了R0切除；且免疫组的ORR和病理完全缓解（pathological complete response, pCR）分别为62.5%和21.9%，均优于化疗组（ORR: 42.9%, pCR: 0%），但差异无统计学意义，可能与化疗组样本量小（*n*=7）有关。此外，与鳞癌和腺癌相比，PLEC的ORR、主要病理缓解（major pathological response, MPR）、pCR均无显著差异，提示肺癌不同组织学亚型可能具有相似的免疫治疗效果。该研究初步证实了新辅助免疫治疗在PLEC中展现出优于新辅助化疗的病理缓解率，且疗效与其他常见NSCLC亚型相当。

Chen等^[62]^的研究进一步支持了上述结论。该研究纳入31例接受新辅助化免联合治疗的II-III期PLEC患者，其中18例（58%）患者实现肿瘤降期，且ORR达到72.4%，8例（25.8%）患者达到了MPR，2例（6.4%）患者达到了pCR。尽管pCR率低于同期分析的鳞癌患者（6.4% *vs* 38.7%, *P*<0.01），但两组DFS无显著差异（17.4 *vs* 15.1个月，*P*=0.54），表明新辅助化免联合治疗可能为PLEC提供与鳞癌相似的生存获益。

上述研究表明，新辅助免疫治疗在PLEC中具有良好疗效，其可安全实现肿瘤降期并提高手术切除率，且生存获益与鳞癌和腺癌相当。然而，由于样本量较小且长期数据缺失，仍需后续研究进一步验证PLEC新辅助免疫治疗的长期疗效。

## 4 总结与展望

PLEC作为一种与EBV感染密切相关的罕见NSCLC亚型，好发于亚洲地区、不吸烟的女性人群。PLEC有着独特的免疫微环境，普遍存在PD-L1高表达且有着丰富肿瘤淋巴细胞浸润，且与EBV的致病机制密切相关。目前，EBV已被广泛视为PLEC疾病进展、预后预测及疗效监测的重要生物标志物，但关于PD-L1表达水平与PLEC预后的关系仍存在一定争议。

目前，晚期PLEC的传统一线治疗以放化疗为主。近年来，免疫治疗在PLEC中的临床探索已取得突破性进展。2021年一项回顾性研究^[59]^首次对比了晚期PLEC患者接受免疫治疗和化疗的疗效，结果显示免疫治疗组的中位PFS显著优于化疗组（15.0 *vs* 7.9个月，*P*=0.005）。后续研究^[30-32,54-58,60]^进一步证实，化免联合治疗相比免疫单药或单纯化疗，在晚期PLEC患者中展现出更优的疗效且安全性良好；新辅助免疫治疗也为可手术患者提供了新的治疗选择。然而，这一领域的深入研究仍面临诸多挑战。

首先，由于PLEC的罕见性，目前报道的研究多为单中心回顾性研究且患者数量较少，易受选择偏倚、混杂变量（如既往治疗差异）及数据完整性不足的影响。例如，2022年Xiao等^[56]^的研究中只详细记录3-4级AEs，未能报道轻度AEs。其次，这些研究中免疫治疗方案和化免联合治疗方案多样，包括帕博利珠单抗、信迪利单抗、纳武利尤单抗等免疫抑制剂联合不同化疗方案，不同治疗方案的疗效可能存在差异，从而难以直接比较具体方案对PLEC患者的疗效，因此需要开展前瞻性队列研究去进一步探讨最佳联合策略。而且，大多数研究的随访时间不足，未能报告成熟的OS数据，期待上述研究长期随访结果的后续更新。此外，目前仍然缺乏高效、特异的PLEC的免疫生物标志物。尽管目前PD-L1和EBV DNA被认为是PLEC的重要生物标志物，但两者均存在不确定性或应用局限性。TMB作为近年来在免疫治疗领域备受关注的潜在生物标志物，部分PLEC研究^[54,55]^认为其在PLEC中的预测价值有限，与患者预后无显著相关性。总体而言，尽管已有研究陆续发现多种潜在标志物，但均缺乏后续前瞻性研究的验证，其临床适用性尚未确立。因此，探寻并验证PLEC特异性的可靠生物标志物，仍是未来的研究重点。最后，免疫治疗的耐药机制亟待阐明。其可能涉及T细胞功能耗竭、免疫抑制细胞浸润、其他免疫检查点的上调，以及EBV相关信号通路的异常激活等。深入理解这些机制有助于开发新的联合治疗策略。

综上所述，免疫治疗（包括晚期患者的化免联合治疗和可手术患者的新辅助免疫治疗）为PLEC患者提供了新的治疗选择，并展现出良好的应用前景。然而，当前证据主要基于回顾性研究，存在样本量小、异质性高等局限性。因此，未来亟需开展多中心前瞻性临床研究去明确免疫治疗在PLEC的疗效与安全性，并探索最佳治疗模式。同时应进一步探索PLEC免疫机制和生物标志物，开发新的治疗方案及寻找能精确预测疗效的生物标志物。
